# O054. Osteopathic manipulative treatment of headache in a polytrauma patient: case report

**DOI:** 10.1186/1129-2377-16-S1-A181

**Published:** 2015-09-28

**Authors:** Vito Adragna, Simona Piazzola, Giacomo Lo Voi

**Affiliations:** Research Department, Study Centre for Traditional Osteopathy, Rome, Italy

## Background

The International Headache Society classification (ICHD-III) ranks among secondary headaches those arising as a result of a head and/or neck injury [1, 2]. To the authors’ knowledge, there are no prior reports in the literature that describe an osteopathic manipulative treatment (OMT) approach for patients with chronic post-traumatic headache.

## Case description

A woman 50 years of age had an automobile accident in 1994 with mild head trauma (GlasgowComaScale=13), signs and symptoms of concessive syndrome; distraction of the cervical spine; fractures of acetabulum, pelvis, femural neck, knee, ulna and radius; dislocated shoulder was hospitalized for surgery. During hospitalization she began to suffer from headaches. A neurological examination and neuroradiological investigation were performed without relief from abnormality. She was then diagnosed with post-traumatic headache. From 1995 to 2005 she underwent surgeries for the removal of fixation and prosthetic hips with rehabilitation. In this period she developed a permanent recurrent headache: two/three attacks per month for a period of two/three days, not always tolerated and treated with ibuprofen. In 2012 tamponade, with a verticalization of the cervical spine. She was prescribed a Shanz collar brace, drug therapy and physiotherapy. Despite the therapy, she continued to have about three episodes of headache per month. In 2013, with a diagnosis of chronic post-traumatic headache attributed to mild head injury (ICHD-III codes: 5.2.2 - ICD-10: G44.31) she came to our consultation. In the previous fortnight she had had continuous headache.

## Description of treatment

The osteopathic manipulative treatment (OMT) was applied individually and different techniques were used depending on the somatic dysfunctions (SD) that were found. Five treatments were performed. The first three, two weeks apart, the fourth after three weeks and the fifth at a distance of one month.

## Results

**Figure 1:**
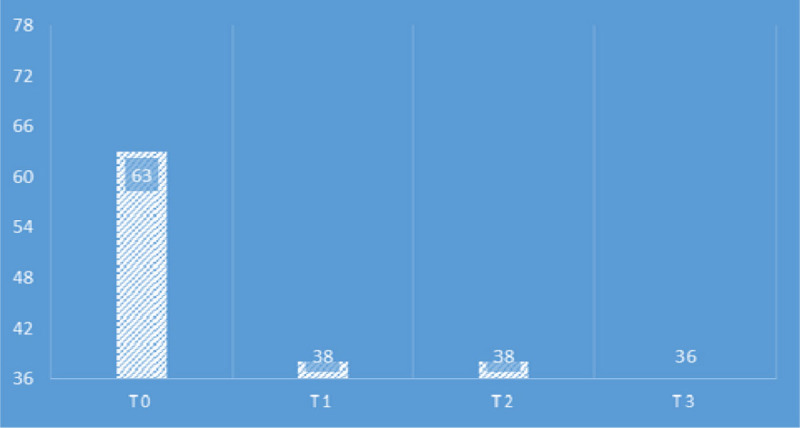
HIT-6 score.

**Figure 2:**
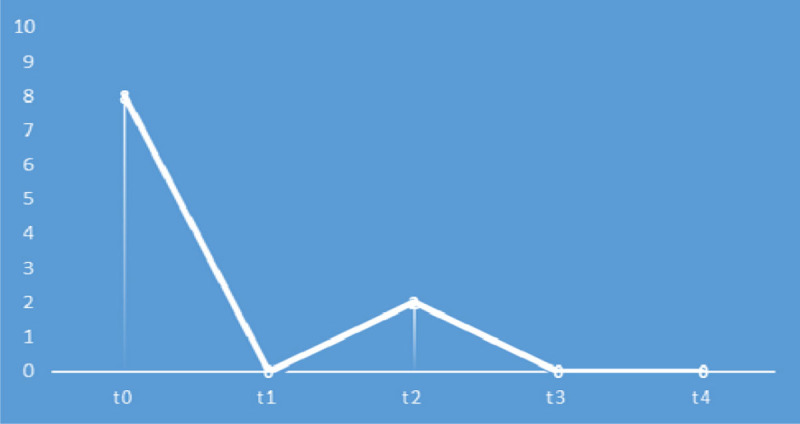
NRS score.

Outcomes were measured by HIT-6 scale at the first (t0) and the last treatment (t1), and then at a distance of one month after the last treatment (t2) and by a scale of quantitative evaluation of pain NSR before each treatment (t0-1-2-3-4). For the HIT-6 (Figure 1) results were: t0=63; t1 and t2=38. For the NRS (Figure 2) results were: t0=8, t1=0, t2=2, t3 and t4=0.

## Conclusions

The OMT was found to have changed the impact of headache on the quality of life of the patient, from important to minimum or no impact at all. One year after the last treatment, the patient reported having had only two episodes of headache but mild, lasting only one day and no longer needed to take medication.

Written informed consent to publish was obtained from the patient(s).
